# Biomechanical Behaviors of Molars Restored with Endocrowns Composed of Different Materials

**DOI:** 10.3390/ma18020250

**Published:** 2025-01-08

**Authors:** Zhi Li, Junxin Zhu, Yongxiang Xu, Xudong Bao, Xiaoyan Wang

**Affiliations:** 1 Department of Cariology and Endodontology, Peking University School and Hospital of Stomatology, Beijing 100101, China; honeys0@163.com; 2 Second Dental Center, Peking University School and Hospital of Stomatology, Beijing 100101, China; dzjx@outlook.com; 3 Department of Dental Materials, Peking University School and Hospital of Stomatology & National Center for Stomatology & National Clinical Research Center for Oral Diseases & National Engineering Research Center of Oral Biomaterials and Digital Medical Devices & Beijing Key Laboratory of Digital Stomatology, Beijing 100081, China; xuyx@hsc.pku.edu.cn

**Keywords:** biomechanical behavior, ceramics, endocrown, fractographic analysis, high-speed camera system

## Abstract

To assess the biomechanical behaviors of endodontically treated molars (ETMs) restored with endocrowns composed of different materials, forty mandibular molars were assigned to five groups (n = 8 each). Untreated molars constituted the control group (group C); the rest of the teeth that underwent root canal therapy were restored with endocrowns composed of polycrystalline ceramics (ST zirconia^®^, UPCERA) in group ZR, lithium disilicate glass ceramics (UP.CAD^®^, UPCERA) in group LD, resin-based nanoceramics (Hyramic^®^, UPCERA) in group NC, and feldspathic ceramics (CEREC Blocs^®^, Sirona) in group FC. All teeth were axially loaded until fracture. The process was recorded using a high-speed camera system, and fractographic analysis was conducted. The results showed that fracture loads did not significantly differ among groups C, LD, and NC; the loads were significantly lower than the load in group ZR but higher than the load in group FC. The mean time from the initial crack to complete tooth fracture varied. Group C had the longest time, followed by group NC; groups ZR, LD, and FC had the shortest time. Similar failure patterns were observed in groups ZR and LD, which were more regular than the pattern in group NC; group FC exhibited the roughest fracture surfaces. Fracture resistance testing combined with a high-speed camera system and fractographic analysis provides a promising modality for studying the biomechanical behaviors of restored teeth. Endocrowns composed of lithium disilicate glass ceramics or resin-based nanoceramics offer alternative restorations for ETMs with extensive coronal loss.

## 1. Introduction

Recent advances in ceramic materials and adhesive technologies have enabled the increasing use of endocrowns to restore endodontically treated posterior teeth with massive coronal loss. Endocrowns were primarily made of feldspathic ceramics initially. However, the risk of repair failure would increase in teeth subjected to abnormal forces (such as bruxism) due to the material’s low strength. In recent years, materials with superior mechanical properties such as lithium disilicate glass ceramics and polycrystalline ceramics have been commonly used to manufacture endocrowns (Table 1) [1]. Restorations with a high elastic modulus are perceived to cause uneven stress distribution, leading to unfavorable outcomes like root fractures and debonding failures. As a result, resin-based nanoceramics is gaining popularity nowadays due to its similar elastic modulus to that of dentin. Some researchers have compared fracture loads among endocrown-restored teeth with different materials and found that teeth repaired with materials displaying low elastic modulus showed better fracture resistance [2,3]. In contrast, other researchers considered that teeth restored with materials displaying high elastic modulus had significantly higher fracture loads [4,5,6]. Thus far, the selection of restorative materials with varying elastic modulus for endocrowns remains a source of disagreement among clinicians.

Most clinical studies have used prospective analysis methods to investigate the repair outcomes for restored teeth [7,8]. Prospective studies have several limitations, including long follow-up periods, high drop-out rates, and multiple influencing factors. In biomechanics research, static analysis using the finite element method is widely used because of its advantages regarding accurate control of variables, as well as its ability to analyze stress distributions within samples [9,10]. However, teeth are subjected to complicated fatigue loads and varied temperatures that are difficult to reproduce through numerical simulation. Therefore, many in vitro studies have used thermal cycling and mechanical loading tests to simulate variations in the mechanical properties of teeth during changes to cyclic load and temperature, along with fracture tests to assess fracture resistance [11,12]. Nevertheless, the stress states and fracture behaviors of the restored teeth remain unclear.

The biomechanical performances of repaired teeth have been recently explored by fractographic analysis based on scanning electron microscopy (SEM) [12,13]. Assessments of characteristic cracks and stress traces on fracture surfaces allow stages of crack propagation to be distinguished, and the features of stress transmission and distribution can be analyzed; thus, the initial locations can be identified, and situations that lead to fracture can be predicted. Because of their ability to rapidly capture high-resolution images, high-speed camera systems have been used for research concerning the use of ultrasound or laser to modify dental hard tissues or remove infections from root canals [14,15,16]. To our knowledge, there has been minimal research involving fracture resistance testing combined with high-speed camera systems and SEM to analyze fracture modes and crack propagation processes in repaired teeth.

This study aimed to explore the effects of restorative material composition on fracture resistance in endodontically treated molars (ETMs) restored with endocrowns using fracture tests combined with a high-speed camera system and fractographic analysis. The null hypothesis was that restorative material composition (including polycrystalline ceramics, lithium disilicate glass ceramics, resin-based nanoceramics, and feldspathic ceramics) would not affect fracture resistance (including fracture loads and fracture characteristics and patterns) in ETMs repaired with endocrowns.

## 2. Materials and Methods

The study was approved by the Institutional Review Board of Peking University School and Hospital of Stomatology (protocol code PKUSSIRB-2024105211) for the year 2024. Forty intact mandibular molars extracted due to periodontitis or pericoronitis at Peking University School and Hospital of Stomatology were collected. They were randomly divided into five groups, each containing eight specimens. The sample size was determined through power analysis with α = 0.05 and a power of 80%. Untreated molars constituted the control group (group C); the remaining teeth received root canal treatment and were then repaired with endocrowns composed of polycrystalline ceramics (ST zirconia^®^; UPCERA, Shenzhen, China) in group ZR, lithium disilicate glass ceramics (UP.CAD^®^; UPCERA, Shenzhen, China) in group LD, resin-based nanoceramics (Hyramic^®^; UPCERA, Shenzhen, China) in group NC, and feldspathic ceramics (CEREC Blocs^®^; Dentsply Sirona, Milford, DE, USA) in group FC. All procedures were carried out by the same skilled dentist to maintain consistency and minimize technical deviations.

### 2.1. Endodontic Procedures

The teeth in group C were untreated; for the remaining teeth, standard root canal treatments were received, and their coronal dental hard tissues were then horizontally removed above the cemento-enamel junction (CEJ) [17]. The dentin surfaces inside the pulp chamber were treated with Clearfil SE Bond adhesive (Kuraray Noritake, Okayama, Japan) following the manufacturer’s instructions. Filtek Z350 XT flowable composite resin (3M ESPE, Saint Paul, MN, USA) was then used to fill concave regions on the sidewalls, and AP-X composite resin (Kuraray, Noritake, Okayama, Japan) served as the base material to maintain an endocrown pulp chamber extension depth of 2 mm. Both composite resins were light-cured (Ivoclar Vivadent, Liechtenstein, Germany) for 20 s at a power density of 1200 mW/cm².

### 2.2. Endocrown Fabrication

As preparation for endocrowns, sidewalls around the pulp chamber were trimmed until the resulting thicknesses were within the range of 2.5 mm to 2.8 mm, and abduction angles were approximately 6° according to the following steps: sidewall thicknesses were measured with a vernier scale before processing, and the regions that should be removed were clearly marked; during endocrown preparation, the sidewalls were trimmed using diamond burs and their thicknesses were measured three times consecutively; after preparation, the sidewall thicknesses were verified both manually using a vernier scale and with 3D scanning for accuracy. The prepared teeth were meticulously refined until the measurements from both methods were totally consistent. All prepared ETMs were scanned with 3Shape TRIOS 2 (3Shape, Copenhagen, Denmark). One ETM was selected as the template, while the remaining 31 were individually shaped to match it in Plasty CAD software (v1.7; 3-DIEMME, Cantù, Italy). The prepared ETM chosen as the template was then restored with an endocrown based on a digital wax pattern created in the 3Shape Dental System software 2018 (3Shape, Copenhagen, Denmark). It was considered as the standardized endocrown. Endocrowns with occlusal morphology identical to the standardized endocrown were fabricated based on the same digital wax pattern to restore the remaining 31 prepared ETMs via the CAD/CAM technique.

The intaglio surface treatments of endocrowns varied according to the materials used. They were etched with 9% hydrofluoric acid (Porcelain Etch; Ultradent, South Jordan, UT, USA) for 20 s in group LD and 60 s in group FC. After etching, the hydrofluoric acid on the intaglio surfaces of endocrowns was thoroughly rinsed off for 60 s. In groups ZR and NC, the intaglio surfaces were sandblasted for 10 s using alumina particles (Ronvig, Daugaard, Denmark) with a size of 50 um or smaller at a blasting pressure of 2–8 kg/cm² (approximately 30–120 psi), with the nozzle positioned 2–10 mm away from the surface. The surfaces were then rinsed with anhydrous ethanol to remove the alumina particles. Subsequently, the dried intaglio surfaces of endocrowns in all groups were treated with 3M ESPE Scotchbond Universal Adhesive (3M ESPE, Saint Paul, MN, USA) for 20 s and gently blown dry.

**Figure 1:**
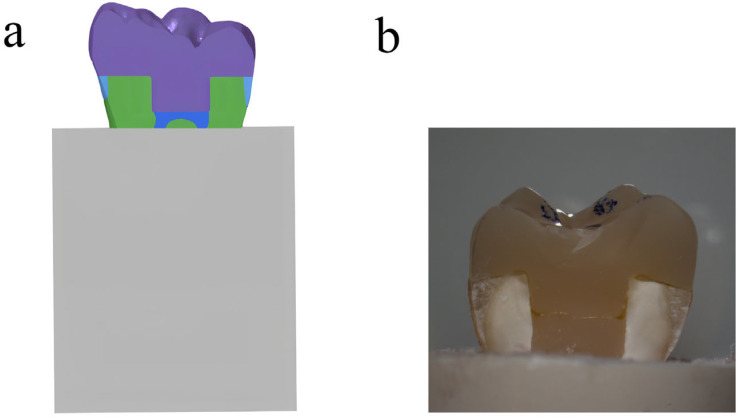
(**a**) Schematic illustration of endocrown-restored molar in bone; (**b**) endocrown-restored molar in sagittal section.

The treatment of tissue surfaces for prepared ETMs was detailed and described in a previous study [17]. In summary, the surfaces were treated with 37% phosphoric acid and then bonded to endocrowns using RelyX U200 dual-curing resin cement (3M ESPE, Saint Paul, MN, USA). A standardized force was applied during cementation by placing a 5 kg weight on the restored teeth for 5 min [18]. Additionally, acrylic resin (Feiying, Zhengzhou, China) surrounded the root portion of restored teeth below the CEJ, as illustrated in Figure 1.

### 2.3. Fracture Resistance Testing

**Figure 2:**
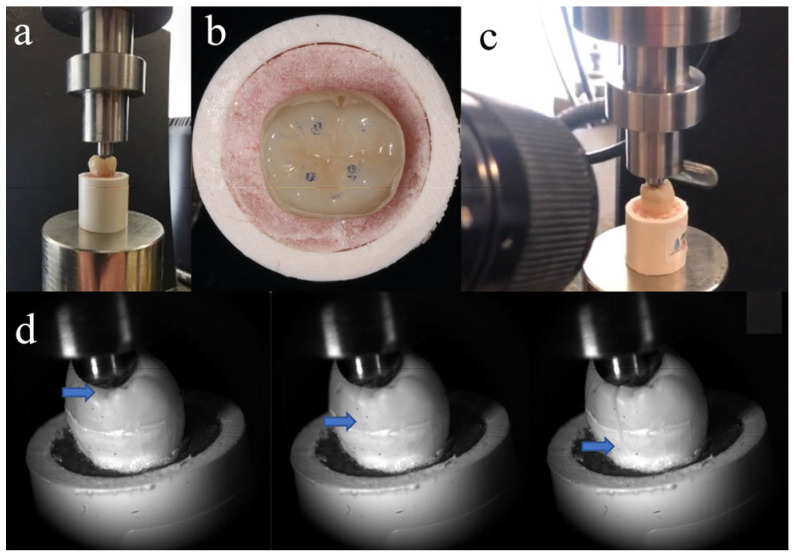
(**a**) Tooth position in universal testing machine; (**b**) loading contact regions between loading head and tooth; (**c**) high-speed camera system used to record fracture process; and (**d**) crack propagation during fracture resistance testing (blue arrow indicates location of crack tip).

Fracture resistance testing was conducted 24 h after preparing the specimens, which were stored in distilled water at 37 °C during this time. As shown in Figure 2a, each tooth was fixed at the same position on a universal testing machine (Instron 5969; Instron, Norwood, MA, USA). To form stable contact, a vertical pre-load of 20 N was initially applied. All endocrowns had identical anatomical morphology, so the loading contact regions were almost consistent (Figure 2b). Molars experience multidirectional forces during chewing. Previous studies have shown that axial load is the primary contributor to the occlusal force in the physiological chewing course [19]. So, a vertical load was applied to the repaired molars (1 mm per minute) until fracture. The loading values at the time of fracture were recorded. The entire process from initial crack formation to complete fracture was recorded using Fastcam SA-Z (Photron, Tokyo, Japan) at 20,000 frames per second (Figure 2c,d). After the fracture occurred, samples were examined using a stereo microscope (SZ-61 Olympus; Olympus Corporation, Tokyo, Japan) at 10× magnification. The fractures were categorized into two types: cracks that terminated above the CEJ indicated repairable fractures, while those that ended below the CEJ were classified as non-repairable fractures. The surface fracture characteristics were observed with a Zeiss EVO 18 SEM (Zeiss, Jena, Germany).

### 2.4. Statistical Analysis

Fracture loads and failure modes were analyzed using IBM SPSS software (v26.0; IBM Corporation, Armonk, NY, USA). Levene’s test was used to assess the homogeneity of variance among groups C, LD, NC, ZR, and FC. A one-way ANOVA was performed to detect the difference in the fracture load among these groups. For the failure modes, Fisher’s exact test was used for the comparison of repairable and non-repairable fractures. *p*-values < 0.05 were deemed statistically significant for all tests.

## 3. Results

Groups C, LD, NC, ZR, and FC had similar discrete states according to Levene’s test (*p* > 0.05). A one-way ANOVA revealed that the mean fracture load was highest in group ZR and was lowest in group FC (*p* < 0.05). As shown in Table 2, no statistical differences were found among groups C, LD, and NC (*p* > 0.05).

The fracture patterns among groups were statistically different according to Fisher’s exact test (*p* < 0.05). Table 3 reveals that in group C, most fractures (six of eight teeth) were repairable. In groups ZR and LD, all fractured teeth were non-repairable; in group NC, one of the eight teeth was repairable. Group FC had the highest percentage of repairable fractures among the endocrown-restored molars.

According to the results of the high-speed camera system, the mean total time from initial crack appearance to complete tooth fracture increased as follows: groups ZR, LD, and FC, shorter than 0.5 × 10^−4^ s; group NC, 0.1 × 10^−3^ s; and group C, 0.2 × 10^−3^ s.

**Figure 3:**
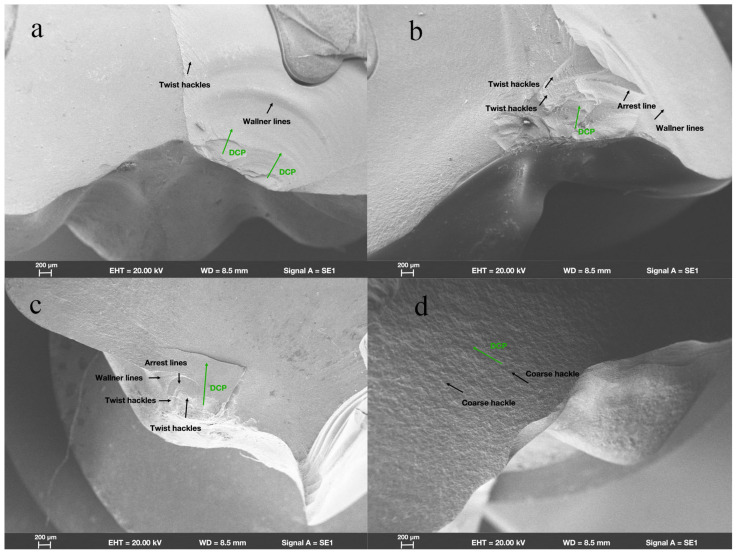
Fractographic analysis of endocrown-restored molars. Scanning electron micrograph images (200×) of endocrowns beneath loading area show characteristics of fracture surfaces and direction of crack propagation (DCP) in groups (**a**) ZR, (**b**) LD, (**c**) NC, and (**d**) FC.

**Figure 4:**
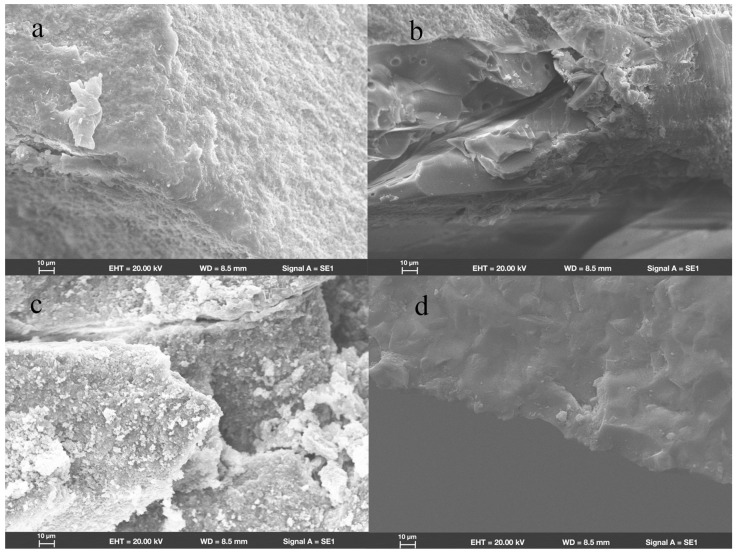
Scanning electron micrograph images (1000×) of endocrowns beneath loading area show intergranular and transgranular fractures in groups (**a**) ZR, (**b**) LD, (**c**) NC, and (**d**) FC.

SEM revealed that in group ZR, fractures originated from the location where the load had been applied. Wallner lines were distributed in a concentric circular pattern adjacent to the fracture initiation site, extending from it in all directions. The fracture surfaces were flat and smooth; few arrest lines and twist hackles were present (Figure 3a). All fracture surfaces mainly comprised intergranular fractures; limited numbers of transgranular fractures appeared in regions near the site of loading head contact (Figure 4a).

In group LD, the fracture initiation sites were also present at locations where the load had been applied. Compared with the fracture characteristics observed in group ZR, group LD exhibited a more irregular distribution of Wallner lines and rougher fracture surfaces; more arrest lines and twist hackles were evident (Figure 3b). More transgranular fractures were observed in regions adjacent to the occlusal surfaces in group LD; as cracks propagated, the number of transgranular fractures decreased, and intergranular fractures constituted most of the fracture surfaces (Figure 4b).

As shown in Figure 3c, the sites of fracture initiation in group NC remained at locations where the load had been applied; however, more fragments were evident nearby. The fracture surfaces in group NC were visibly rougher than the surfaces in groups ZR and LD; group NC also displayed large numbers of arrest lines and twist lines. Similar to the findings in groups ZR and LD, the fracture surfaces mainly exhibited intergranular fractures, along with a few transgranular fractures (Figure 4c).

Among all the teeth in the present study, the fracture surfaces in group FC were the roughest. Coarse hackles, which indicate poor material strength, were only evident in group FC (Figure 3d). Although intergranular fractures remained the dominant type on fracture surfaces, the number of transgranular fractures was higher than the numbers in other groups (Figure 4d).

## 4. Discussion

Endocrowns have been regarded as optional restorations for ETMs with extensive coronal loss [1,20,21]. Some studies considered that endocrown-restored teeth using different materials exhibited similar fracture resistance, while others did not [1,2,3]. Thus far, there remains no consensus regarding the selection of materials used to fabricate endocrowns. High-speed camera systems can capture images at tens of thousands of frames per second, allowing for a detailed recording of the process from crack formation to tooth fracture. By analyzing the timing of crack propagation within teeth along with fractographic analysis, potential fracture mechanisms for restored teeth can be inferred. To the authors’ knowledge, there is limited research studying the biomechanical behaviors of teeth using high-speed camera systems. The present study investigated the effect of material composition on fracture resistance in endocrown-restored molars using fracture tests combined with a high-speed camera system and fractographic analysis and found that the fracture loads and fracture characteristics in restored teeth differed according to endocrown composition. Thus, the null hypothesis—that restorative material composition does not affect the fracture resistance of endocrown-restored molars—was rejected.

Polycrystalline ceramics, such as zirconia materials, are commonly used to fabricate prostheses because of their excellent mechanical properties. The present study showed that the mean fracture load of ETMs restored with zirconia endocrowns was significantly higher than those of intact molars and ETMs restored with endocrowns composed of other materials. This result is consistent with the findings in previous studies [4,18]. It may be attributed to the transformation toughening mechanisms in zirconia, which can be classified as 3Y-TZP ceramics in the current study. The toughening mechanism refers to how materials enhance their resistance to crack propagation. Zirconia includes monoclinic, cubic, and tetragonal forms. When cracks undergo internal propagation, tetragonal-phase crystals within the stress field around the crack tip transform into a monoclinic phase, leading to increased crystal size. This change creates compressive stress near the crack tip, thereby hindering the crack from advancing via energy consumption [22]. The present study also demonstrated that fractures in molars restored using zirconia endocrowns were usually non-repairable, exhibiting few arrest lines and twist hackles on the fracture surfaces. This phenomenon occurs because zirconia undergoes volume shrinkage after sintering, leading to a uniform and dense crystal arrangement. A high driving force is required because of the dense crystal arrangement and transformation toughening mechanisms in zirconia; however, once the external driving force exceeds the fracture strength, cracks encounter minimal resistance in zirconia and tend to propagate apically without obvious deflection in restored teeth; they typically extend beneath the CEJ. Therefore, ETMs restored with zirconia endocrowns have high fracture strength, but fractures that develop in such teeth are usually non-repairable [4]. Additionally, zirconia is challenging to etch, leading to inferior bonding compared to etchable ceramics. Therefore, routine use of zirconia endocrowns is not recommended. However, its high fracture strength makes it suitable for restoring ETMs with limited occlusal–gingival distance.

Lithium disilicate glass ceramics are commonly used to fabricate restorations due to good mechanical properties and excellent aesthetic performance. This study found that lithium disilicate glass ceramics resulted in lower fracture loads for ETMs restored with endocrowns compared to zirconia ceramics. However, fracture loads were similar for intact molars and those restored with glass ceramic endocrowns, suggesting that the endocrown-restored molars using glass ceramic could partially regain normal physiological function. The crystal structure of lithium disilicate glass ceramics consists of numerous interlocking needle-like crystals embedded in the glass matrix. This configuration leads to a rougher fracture surface with more arrest lines and twist hackles than that of zirconia. Our study also suggested that fractures in the repaired molars were usually non-repairable. Both the fracture time and SEM findings suggested that fracture behaviors were similar for ETMs restored with endocrowns composed of lithium disilicate glass ceramics or zirconia ceramics. Once the external driving force exceeded the fracture strength, cracks propagated in the glass ceramic endocrowns with minimal deflection, following a nearly straight path. Therefore, cracks tended to reach locations below the CEJ, resulting in non-repairable fractures. Men have an average maximum bite force of about 630 N, while women’s average is around 430 N. The mean fracture load of endocrown-restored molars made of glass ceramics is approximately 3900 N, which far exceeds the maximum bite force in the molar region during normal occlusion [23]. Furthermore, retention loss is the primary cause of endocrown failure. The acid-etchable characteristics of lithium disilicate glass ceramics result in superior bonding to dental hard tissues, compared with zirconia ceramics. Therefore, lithium disilicate glass ceramics can be regarded as appropriate restorative materials for ETMs with extensive coronal loss [6].

In recent years, resin-based nanoceramics have been increasingly used for endocrown fabrication because of their similar elastic modulus to dentin and good aesthetic properties [7]. Intriguingly, although the strength of nanoceramics is much lower, fracture loads do not significantly differ between ETMs restored with nanoceramic and lithium disilicate glass ceramic endocrowns. The result is consistent with previous findings [24]. For ETMs restored with nanoceramic endocrowns, the total time from initiation of cracking to complete fracture was 0.1 × 10^−3^ s, which was much longer than the time among ETMs restored with glass ceramic endocrowns (shorter than 0.5 × 10^−4^ s). This result indicated that crack propagation in nanoceramics encountered greater resistance. Compared with glass ceramics, resin-based nanoceramics exhibited more arrest lines and twist hackles on fracture surfaces, suggesting that certain factors hindered crack propagation and caused a change in the stress state; consequently, the principal stress axis rotated or twisted, further altering the direction of crack growth and disrupting crack propagation. Based on SEM observations in combination with a high-speed camera system, we speculated that the following factors contributed to its good fracture resistance: resin-based nanoceramics are generally composed of resin polymers and inorganic fillers. Resin polymers are relatively soft, allowing them to dissipate energy by deforming during crack propagation. Additionally, cracks tend to deflect towards softer resin polymers when crack tips reach stiffer inorganic fillers; the process of crack deflection, which alters the original path and generates non-planar cracks, could also dissipate energy. Therefore, cracks in nanoceramics can only propagate through increasing driving force, leading to good fracture resistance in restored molars. The proportions of resin polymers in nanoceramics differ among commercially available products. These differences may explain why some studies showed similar fracture resistance in molars restored with endocrowns composed of nanoceramics relative to ETMs restored with glass ceramics, whereas other studies showed the opposite result [5,24]. Unlike the propagation in zirconia and glass ceramics, cracks in resin-based nanoceramics encounter obstacles more frequently and are likely to change directions during propagation; accordingly, cracks spread in multiple directions rather than directly towards the tooth root. Additionally, with the reason that the elastic modulus of resin-based nanoceramic is similar to that of dentin, stress distributions in the cervical area of molars restored with nanoceramic endocrowns are more even than those restored with glass ceramic endocrowns. The above factors increase the likelihood of repairable fractures. In molars restored using resin-based nanoceramic endocrowns, most fractures remain non-repairable; however, the incidence of repairable fractures is higher than the incidences in molars restored with zirconia or lithium disilicate glass ceramic endocrowns [2,3,5]. For restored teeth with cracks extending above the CEJ, additional prosthodontic treatments are possible. According to the present study, resin-based nanoceramics can be considered as an alternative material for manufacturing endocrowns. Although thermal cycling is considered to negatively impact the strength of ceramics in the oral environment, the extent of strength reduction for various ceramics remains controversial. Therefore, long-term clinical research is still necessary to evaluate the clinical efficacy of endocrown-restored molars using nanoceramics.

Feldspathic ceramics were commonly used to fabricate endocrowns in past decades [25,26]. In recent years, these materials have been used with less frequency because of their low strength and poor mechanical stability. The present study demonstrated that ETMs restored with feldspathic ceramic endocrowns had the lowest fracture loads compared with ETMs restored with other materials, which was consistent with a previous study [27]. SEM revealed the presence of coarse hackles on fracture surfaces, indicating that feldspathic ceramics had low strength and toughness. As a result, molars restored with endocrowns composed of feldspathic ceramics are susceptible to fracture before crack propagation to a location below the CEJ, increasing the likelihood of repairable fracture. Therefore, the incidence of repairable fractures in molars restored using feldspathic ceramic endocrowns is much higher than the incidences in teeth restored with polycrystalline ceramics, lithium disilicate glass ceramics, or resin-based nanoceramics [28,29].

Teeth are subjected to fatigue loads and temperature changes in the oral environment [30,31], which in turn may affect crack propagation. Lateral force is considered more dangerous to teeth than axial load, increasing the risk of fracture. Therefore, further research including thermo-mechanical cyclic loading in different directions is suggested to better mimic the oral environment. Additionally, the present study used a high-speed camera system in combination with SEM to observe complete fracture surfaces and compare fracture characteristics among various materials. Crack propagation behaviors and toughening effects at various stages in the repaired teeth, and the impact of materials’ elastic modulus on stress distribution at the tooth-restoration interface require further investigation. Furthermore, X-ray diffraction is recommended to confirm the tetragonal-to-monoclinic transformation of zirconia during the fracture process.

## 5. Conclusions

Within the limitations of this study, the following conclusions can be drawn:

Fracture resistance testing combined with a high-speed camera system and fractographic analysis provides a promising modality for studying the biomechanical behaviors of restored teeth. The fracture loads and failure patterns of restored teeth can be affected by the structural characteristics of restorative materials. Endocrowns composed of lithium disilicate glass ceramics or resin-based nanoceramics can be regarded as optional restorations for ETMs with extensive coronal loss.

## Figures and Tables

**Table 1:** Material type, composition, and properties.

Materials	Type	Composition	Elastic Modulus (GPa)	Flexural Strength (MPa)
CEREC blocs^®^	feldspathic ceramics	SiO_2_ 56–64%, Al_2_O_3_ 20–23%, Na_2_O 6–9%, K_2_O 6–8%, CaO 0.3–0.6%, TiO_2_ 0–0.1%	45.0	154.0
UP.CAD^®^	lithium disilicate glass ceramics	SiO_2_ 58.5–72.5%, Li_2_O 13–15%, K_2_O 3–5%	95.0	400.0
ST zirconia^®^	polycrystalline ceramics	Nanometer-zirconia powder > 98%, Fe_2_O_3_ < 0.3%, Pr_2_O_3_ < 0.2%, Er_2_O_3_ < 0.1%	220.0	1100.0
Hyramic^®^	resin-based nanoceramics	Polymerized resin 13–43%, inorganic filler 55–85%	15.0	180.0

Provided by manufacturer.

**Table 2:** Fracture loads (mean ± standard deviation) among groups C, LD, NC, ZR, and FC.

Group	Mean ± SD (N)
C	3069.34 ^a^ ± 939.50
LD	3931.63 ^a^ ± 1367.44
NC	3537.18 ^a^ ± 763.65
ZR	5321.56 ^b^ ± 1410.88
FC	1799.62 ^c^ ± 986.72

SD, standard deviation. ^a,b,c^ different superscript letters indicate significant differences between groups (*p* < 0.05).

**Table 3:** Numbers of repairable and non-repairable fractures in groups C, ZR, LD, NC, and FC.

Group	Repairable Fractures	Non-Repairable Fractures
C	6	2
ZR	0	8
LD	0	8
NC	1	7
FC	3	5

## Data Availability

Data are contained within the article.
